# Effect of a Gluten-Free Diet on Whole Gut Transit Time in Celiac Disease (CD) and Non-Celiac Gluten Sensitivity (NCGS) Patients: A Study Using the Wireless Motility Capsule (WMC)

**DOI:** 10.3390/jcm13061716

**Published:** 2024-03-16

**Authors:** Orestes Cobos-Quevedo, Gildardo Alfonso Hernández, Xaira Jimena Rivera-Gutiérrez, Peter Grube-Pagola, José María Remes-Troche

**Affiliations:** Instituto de Investigaciones Médico-Biológicas, Universidad Veracruzana, Veracruz 91400, Mexico; orestes.cq7@gmail.com (O.C.-Q.); dr.gildardo.hernandez@gmail.com (G.A.H.); xaira-rivera@ibt.unam.mx (X.J.R.-G.); pgrube@uv.mx (P.G.-P.)

**Keywords:** celiac disease, gluten, diet, non-celiac sensitivity, wheat

## Abstract

**Background**: This study explores the impact of a gluten-free diet (GFD) on regional gastrointestinal (GI) transit times in individuals with celiac disease (CD) and non-celiac gluten sensitivity (NCGS). While a GFD is established for managing CD and wheat allergy, its effects on NCGS remain controversial due to inconclusive evidence. **Methods:** Utilizing a wireless motility and pH capsule (WMC) to assess regional (measurements of gastric, small bowel, and colonic transit times) and whole gut transit, newly diagnosed CD (*n* = 12) and NCGS (*n* = 12) patients underwent evaluations at baseline and 4 weeks after having a GFD. **Results**: At baseline conditions, individuals diagnosed with CD exhibited prolonged colonic and intestinal transit times when compared to those with NCGS (*p* < 0.05). Following a 4-week GFD, CD patients experienced significant reductions in both intestinal and colonic transit times, along with enhanced small intestine contractility. NCGS individuals showed improvements in intestinal transit time and contractility with a GFD, although the colon exhibited no discernible effect. The GFD did not significantly impact intragastric, intestinal, or colonic pH. **Conclusions:** This study, employing WMC for the first time, provides novel insights into the positive effects of a GFD on intestinal and colonic transit, as well as contractility, in CD patients, and to a lesser extent, in those with NCGS.

## 1. Introduction

Gluten-related disorders constitute a spectrum of conditions triggered by the ingestion of gluten, a protein found in wheat, barley, and rye. Celiac disease (CD) is the most well-known and extensively studied gluten-related disorder [1]. It is estimated to affect approximately 1% of the global population, although prevalence rates vary significantly across different regions and populations [2,3,4]. CD is more common in individuals of European descent but can occur in people of any ethnicity [2]. There has been a notable increase in the prevalence of CD over the past few decades, likely due to increased awareness, improved diagnostic techniques, and changes in environmental factors such as dietary habits and gluten consumption [2,3].

Non-celiac gluten sensitivity (NCGS) is another important entity within the spectrum of gluten-related disorders [1]. Unlike CD, NCGS does not involve autoimmune mechanisms or intestinal damage [2,5,6]. While the precise mechanism remains unclear, it is suggested that the innate immune response, rather than the adaptive immune system, may be involved in NCGS [7,8]. The prevalence of NCGS is less well-defined compared to CD, partly due to challenges in diagnosis and varying diagnostic criteria. Estimates suggest that NCGS may affect a significant proportion of the population, although precise prevalence rates remain uncertain. NCGS can occur in individuals of any age, gender, or ethnicity and is often diagnosed in adults experiencing unexplained gastrointestinal symptoms after gluten consumption.

Both CD and NCGS patients commonly experience gastrointestinal symptoms, ranging from abdominal discomfort to irregular bowel habits, necessitating a meticulous examination of the factors influencing, such as motility abnormalities. Dysmotility in the gastrointestinal (GI) tract has been described in patients with CD and may be linked to the generation of symptoms [9,10,11,12]. Some of the mechanisms associated with dysmotility in gluten-related disorders include impaired food absorption, low-grade inflammation in the small intestine, dysfunction of the autonomic nervous system, hormonal imbalances, and dysbiosis [13]. In fact, it has been described that a gluten-free diet (GFD) can reverse motor disturbances in patients with CD, but its effect on NCGS is unknown [11,13,14]. It is important to highlight that the few studies that have assessed motor alterations in the small intestine and colon in patients with CD have used tests such as small intestine manometry and oro-cecal transit [9,10,11,12,13,14,15].

Prior to the introduction of the wireless motility capsule (WMC) (SmartPill^®^, Given Imaging, Yoqneam, Israel), evaluating the complete transit profile of the gut in a single test without the use of radiation posed significant challenges. The WMC presents a groundbreaking method capable of assessing the entire gut transit time (WGTT) and regional transit times, including gastric emptying time (GET), small bowel transit time (SBTT), and colonic transit time (CTT) [16]. This capsule, measuring 26.8 mm in length and 11.7 mm in diameter, is equipped with sensors for temperature (ranging between 25 and 49 °C), pH (ranging between 0.05 and 9.0), and pressure (ranging between 0 and 350 mmHg) [17]. While navigating through the gastrointestinal tract, the capsule continuously records various measurements and wirelessly transmits real-time data to a receiver worn by the patient on their waist throughout the study. While numerous studies have explored the utility of WMC in identifying multiregional dysmotility in various conditions (such as diabetes [18], chronic constipation [19] and gastroparesis [20]), no current data are available regarding the GI tract transit profile among patients with CD and other gluten-related disorders, such as NCGS.

While the implementation of a GFD has become a cornerstone in managing CD and NCGS, providing a therapeutic avenue to alleviate symptoms and prevent long-term complications, the specific influence of gluten withdrawal on WGTT warrants a more in-depth investigation. This study seeks to bridge this knowledge gap by employing the WMC, a non-invasive and patient-friendly technology, to monitor real-time transit dynamics in response to dietary modifications.

## 2. Materials and Methods

### 2.1. Patients

A prospective observational study was conducted (between January and December 2018), recruiting patients (*n* = 42) consecutively from the gastroenterology department of our hospital if they presented symptoms suggestive of either CD or NCGS. Patients with a confirmed diagnosis of CD or NCGS who were already adhering to a gluten-free diet (GFD) were excluded (*n* = 18). CD (*n* = 12) diagnosis was established when initial evaluations yielded positive results for IgA tissue transglutaminase antibodies (IgA-tTG2) and/or IgG deaminated gliadin peptide antibodies (IgG-DGP), along with the presence of HLA-DQ2 and/or DQ8 haplotypes, and intestinal villous atrophy observed in duodenal biopsies according to the Marsh–Oberhüber classification [21]. NCGS was suspected in subjects with negative serology and biopsy results but reported symptoms associated with gluten consumption. Both a gastroenterologist and a nutritionist evaluated these subjects, analyzing gastrointestinal and/or extraintestinal symptoms (such as abdominal bloating, flatulence, changes in bowel habits, fatigue, headache, and muscle pain) and their correlation with gluten intake using the GSRS questionnaire [22]. The GSRS questionnaire has been previously employed to assess digestive symptoms and extraintestinal manifestations in NCGS patients, recommended by the Salerno consensus [5]. All subjects were prescribed a GFD by a nutritionist, and adherence was monitored through food diaries and weekly supervision for at least 6 weeks. NCGS diagnosis was confirmed if patients experienced a reduction in more than 30% in at least three of the symptoms assessed by the GSRS for at least half of the observation period (during at least three of the six weekly evaluations). To confirm NCGS, all subjects underwent a gluten challenge for two weeks (prescribed by the nutritionist, with a recommendation to consume at least 8 g of gluten daily). NCGS diagnosis was corroborated when patients (*n* = 12) reported the recurrence of previously evaluated symptoms. Following the gluten challenge phase of the study, patients returned to a GFD. Stool frequency was also assessed using the Bristol stool form scale [23].

### 2.2. Baseline Evaluation

A baseline clinical evaluation and physical examination were performed on all the subjects. All the patients underwent the following assessments (routinely requested by the nutrition service): body mass index (BMI) (kg/m^2^), and blood pressure (mmHg), as well as the determination of glycemia (mg/dL), total cholesterol (mg/dL), HDL (mg/dL), and triglycerides (mg/dL).

#### Wireless Motility Capsule

All patients undergoing WMC testing at our institution adhere to a standardized protocol as previously outlined [23]. They are advised to discontinue proton pump inhibitors for 7 days, histamine-2-receptor antagonists for 3 days, and antacids for 1 day before ingesting the WMC. Additionally, patients are instructed to cease the use of prokinetic, antiemetic, anticholinergic, antidiarrheal, narcotic pain, and non-steroidal anti-inflammatory drugs for 3 days prior to the test. On the day of the procedure, following an overnight fast, patients consume a standardized meal consisting of a nutrient bar (Smartbar^®^, Given Imaging, Mansfield, MA, USA) and 50 cc of water before ingesting the WMC. Subsequently, patients fast for 6 h after ingesting the capsule, with small sips of water permitted. Throughout the study, patients are required to wear a data receiver within five feet of their body at all times. This receiver, measuring 6″ × 4″ × 1.5″ and equipped with a rechargeable battery, records data and prompts patients to log certain events, such as food intake, bowel movements, and sleep, by pushing the event button and maintaining a diary. Patients are instructed to avoid alcohol, laxatives, antidiarrheal medications, or initiating new medications (unless necessary) until the capsule is expelled. Upon completion of the study, the receiver is returned, and data are analyzed based on pH and temperature fluctuations. Gastric emptying time (GET) is considered delayed if it exceeds 5 h, defined as the duration from ingestion until the capsule reaches the duodenum (marked by a pH increase in more than 3 units). Small bowel transit time (SBTT), the duration from the duodenum to the cecum, is deemed normal when ranging between 2.5 and 6 h, identified by a sudden pH drop of more than 1 unit sustained for at least 30 min. Colonic transit time (CTT), from cecal entry to capsule expulsion, is classified as delayed if surpassing 59 h, determined by a sudden temperature drop or signal loss. Whole gut transit time (WGTT), the sum of transit times through all segments, is considered normal if less than 73 h. Motility and contractility measures were calculated using 2 pieces of proprietary software (motiligi, version 3.0.20, and GIMS Data Viewer, version 3.0.0, Medtronic, Minneapolis, MN, USA). MotiliGI—the contractility and motility measures provided by the software include mean peak amplitude of contractions, contractions per minute and the motility index (MI) in the small bowel [24]. Motility index is a composite parameter that incorporates both contraction frequency and amplitude and is calculated as ln (sum of amplitudes × number of contractions + 1) assessed on contractions >10 mmHg, but <300 mmHg [25]. It has previously been demonstrated as a useful summary measure, facilitating both a chronotropic and ionotropic assessments of GI motility, although its exact interpretation remains to be fully determined. Also, median pH along segments was calculated.

### 2.3. Intervention

All participants were provided with guidelines for adhering to a gluten-free diet (GFD) as prescribed by a nutritionist, as detailed earlier in this study. Subsequently, they attended weekly appointments for four consecutive weeks (one month). Adherence to the diet was assessed using a questionnaire that captured the foods consumed by the patient during the week leading up to each visit.

### 2.4. Final Evaluation

At the end of the 4 weeks, GSRS questionnaires and BSFS were applied. WMC was repeated to compare motility parameters before and after the intervention.

### 2.5. Statistical Analysis

Descriptive statistics are presented as follows: mean (standard deviation) for normally distributed continuous variables, median (range) for non-normally distributed continuous variables, and number (percentage) for categorical variables. Group comparisons were conducted using the chi-square test, Mann–Whitney U test, and the Wilcoxon signed rank test, as appropriate. Statistical significance was defined as *p* < 0.05 for all differences. Data analysis was performed using SPSS version 21.0 (SPSS Inc., Chicago, IL, USA). Informed consent was obtained from all participants, and the study was approved by the institutional ethics committee. Funding was provided by a grant from CONACYT (FOSIS 2015-262023).

## 3. Results

### 3.1. Demographic and Clinical Data

We assessed 12 individuals with CD (mean age 39 ± 12 years, 11 females, mean BMI 21.2 ± 1.9 kg/m^2^) and 12 with NCGS (mean age 31 ± 5 years, 10 females, mean BMI 23.8 ± 2.1 kg/m^2^) (Table 1). The most common symptoms in patients with CD were bloating (12/12), abdominal pain (10/12), frequent bowel movements (8/12), nausea (6/12), borborygmi (5/12), diarrhea (4/12), and constipation (2/12). On the other hand, in patients with non-gluten celiac sensitivity, the most frequent symptoms were abdominal pain (12/12), bloating (8/12), headache (7/12), muscle pains (6/12), fogginess (4/12), and constipation (3/12).

Among the CD patients, 92% reported over 50% improvement in abdominal pain/distension at the onset of the GFD, while symptomatic improvement occurred in 67% of NCGS patients. In CD patients, there was a significant change in the Bristol Stool Form Scale (BSFS) at the conclusion of the GFD (median basal BSFS 5 versus median final BSFS 3, *p* = 0.02). Conversely, NCGS patients showed no difference in the consistency of bowel movements based on the BSFS before and after GFD (baseline median 3 versus final median 3, *p* = 0.99).

### 3.2. Motility Parameters

Table 2 displays variations in gastrointestinal transit times, both overall and regionally, between individuals with CD and those with non-celiac gluten sensitivity NCGS at the beginning and following four weeks of a GFD.

**Figure 1:**
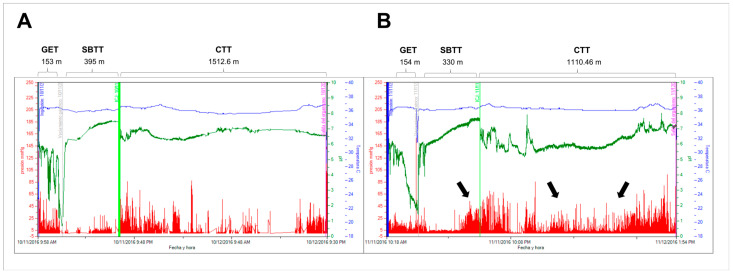
Determination of landmarks and regional transit times on plot data obtained from a WMC recording in a celiac disease patient before (**A**) and after 4 weeks of a gluten-free diet (**B**). Blue line: temperature; white line, pH: red line, pressure. The black arrows in panel B illustrate an evident increase in the motor activity of the small intestine (especially ileum) and colon after the gluten-free diet. GET: gastric emptying time; SBTT: small bowel transit time; CTT: colonic transit time.

Under baseline conditions, individuals diagnosed with CD exhibited prolonged colonic and intestinal transit times when compared to those with NCGS (*p* < 0.05, Table 2). Following a 4-week GFD diet in CD patients, there was a notable reduction in both intestinal and colonic transit times (Table 2, Figure 1). Additionally, GFD augmented small intestine contractility, as evidenced by increased numbers of contractions, greater amplitude, elevated pressure, and a heightened MI (Table 2). At the colonic level, GFD led to an increment in both the number of contractions and pressure (Table 2).

In individuals with NCGS, the GFD improved both intestinal transit time and contractility, but it had no discernible effect on the colon, as observed in Table 1. The gluten-free diet did not exhibit any statistically significant impact on intragastric, intestinal, or colonic pH.

## 4. Discussion

In recent times, there has been heightened scrutiny of the management approaches for CD and NCGS particularly with a focus on dietary interventions. A GFD is the cornerstone of management for patients with CD and NCGS, as it effectively alleviates symptoms and prevents complications associated with gluten ingestion [26,27]. The mechanism underlying the symptomatic improvement on a GFD differs between CD and NCGS, reflecting the distinct pathophysiological processes involved in each condition [1,3,4]. By eliminating gluten from the diet, individuals with CD can effectively prevent the activation of the immune response and subsequent intestinal damage. The improvement in symptoms on a GFD is attributed to the resolution of inflammation and the restoration of intestinal mucosal architecture. Similarly, patients with NCGS experience symptomatic relief on a GFD, despite the absence of autoimmune-mediated intestinal damage characteristic of CD. The exact mechanism underlying NCGS is not fully understood, but it is believed to involve a combination of innate and adaptive immune responses, as well as non-immune-mediated factors [3].

In addition to the anti-inflammatory and immunological effects of a GFD, a pivotal area of investigation revolves around understanding the impact of such diet on the gastrointestinal dynamics of individuals grappling with CD and NCGS. In this research, we explored the intricate connection between gluten consumption and WGTT by utilizing the state-of-the-art WMC technology. The WMC offers an office-based, radiation-free, standardized testing method capable of measuring gastric emptying time, small bowel transit time, and colon transit time simultaneously. Studies have shown that the WMC yields results comparable to traditional motility testing methods involving radiolabeled substances and radiography [17]. Therefore, the WMC should be regarded as an alternative for transit testing in suspected cases of gastroparesis, small bowel dysmotility, and colon transit abnormalities. Additionally, it should be considered the preferred test for suspected conditions involving motility disorders affecting multiple regions or generalized motility issues [17,18,19,20].

Our findings are novel in several ways, as we employed precise definitions (Oslo criteria for celiac disease, Salerno criteria for non-gluten celiac sensitivity), innovative technology (WMC), and an ideal therapeutic intervention (gluten-free diet). Through this approach, we demonstrated that gluten induces colonic intestinal dysmotility, especially in patients with CD. Intestinal dysmotility can develop in numerous conditions where the gut’s ability to regulate muscular activity is compromised due to internal or external factors [9,13]. These conditions, which can be either primary or secondary, often exhibit diverse symptoms such as abdominal distension, recurring blockages, colicky abdominal pain, constipation, gastroesophageal reflux disease, and frequent vomiting. Essentially, any disruption in the movement of food and secretions through the digestive tract can be classified as an intestinal motility disorder.

Previous studies employing various methods such as mouth-to-cecum transit time, lactulose H_2_ breath test, antroduodenal manometry, ultrasound, colonic transit time with radiopaque markers, and ^13^C-occtanoid breath test have reported motor disturbances in the intestine and colon of patients with celiac disease [9,13]. However, these abnormalities have been not appropriately in NCGS.

In our study we found that compared to NCGS, CD exhibited prolonged colonic and intestinal transit times. These results are like those reported by Chiarioni [15] and Benini [14], where it is described that patients with celiac disease have a prolonged oro-cecal transit time when compared to a control group. On the other hand, in another study conducted by Bai and colleagues [28] using radiopaque markers, it was demonstrated that patients with CD have an accelerated colonic transit. The differences between studies may be explained using different methodologies and populations with more severe symptoms (e.g., patients with chronic diarrhea). In our patients with CD, the predominant symptoms were abdominal pain and distension, while only 4 out of 12 experienced diarrhea. Although the classic presentation of CD is more common in young children, consisting primarily of gastrointestinal symptoms with malabsorption (chronic diarrhea, abdominal pain, distension, and failure to thrive or weight loss), some patients also present with constipation [29]. In adults, the presentation of CD is often more subtle and can be mistaken for irritable bowel syndrome, as in our cases [30,31,32].

Regarding the evidence of dysmotility in the small intestine, it has been reported that individuals with CD exhibit fasting motor abnormalities, including clustered contractions, giant jejunal contractions, and bursts of non-propagated contractions, observed in both adults and children with CD [9,10,11]. In a further study, Bassotti et al. [12] validated that most of untreated celiac patients displayed distinct motor abnormalities in the upper gut during both fasting and fed periods. Although it is not precisely understood why gluten induces dysmotility, multiple mechanisms have been proposed, including low-grade inflammation, diminished food absorption, dysfunction of the autonomic nervous system, hormonal imbalances, and dysbiosis [9,13]. Dysmotility associated with CD is not limited solely to the intestine; it has also been described at the level of the gallbladder. For instance, Das et al. [33], using ultrasound (USG) and hepatobiliary scintigraphy (HBS) with mebrofenin labeled with technetium-99, reported that up to 16% of children with CD have impaired gallbladder function, which is reversible once gluten is removed from the diet.

What is clear, according to our results, is that a GFD has a positive effect on symptoms and reverses dysmotility in patients with CD, especially in the small intestine. Significantly, the frequency, amplitude, and pressure of the small intestine, measured by WMC, improved after the intestine was no longer exposed to gluten. Some previous studies have had examined the impact of a GFD on gastrointestinal motility. As in our study, there is evidence that dysmotility could be reversible with a GFD. For example, Cucciara et al. [11] found that gut dysmotilities (reduced postprandial antral motility index and abnormal fasting and fed motor responses) disappeared in children with CD after GFD. In another study, through the use of the oro-cecal transit test with lactulose, it was demonstrated that after a GFD period, the mouth-to-cecum transit time in patients was significantly reduced compared to pre-diet transit (134 +/− 8 vs. 243 +/− 10 min, *p* = 0.0001) and did not show a statistical difference when compared to that found in controls (*p* = 0.1) [15]. Benini et al. [31] showed that following mucosal recovery, the gastric emptying of a gluten-free meal improves, although it remains delayed in comparison to controls. Therefore, it is possible that the improvement in the motor function of the small intestine observed after a gluten-free diet is the result of inflammation resolution.

NCGS is a clinical entity characterized by the absence of CD and wheat allergy in patients that trigger reproducible symptomatic responses to gluten-containing foods consumption [34,35]. The absence of a clear definition, coupled with controversies in clinical trials, indicates a limited understanding of the etiopathogenesis and, besides gluten, other components such as fructans or protein alpha-amylase-trypsin inhibitors. In this population, our study demonstrates for first time that a GFD also has a positive effect on intestinal motility. There is only one previous study that shows that patients with NCGS, diagnosed by gluten-related symptoms and presence of IgG AGA, present with motility alterations that improve in most cases after a GFD [30]. Although an exact mechanism is yet to be elucidated, it is hypothesized that the innate but not adaptive immune response may play a part in NCGS [5,6,7,34]. Suggested factors encompass alterations in intestinal permeability, abnormal motility, and gut stimulation. In specific animal models, like mice expressing human leukocyte antigen (HLA)-DQ8 genes, gliadin has been proposed to induce hypercontractility of smooth muscle and dysfunction in cholinergic nerves, all without causing atrophy in the duodenal mucosa [36,37]. Studies involving mice indicate that wheat germ agglutinins provoke the release of IL-4 and IL-13, elevate inflammation, disrupt epithelial integrity, and enhance the synthesis of proinflammatory cytokines [38]. Therefore, just as it is described that inflammation and epithelial alterations are associated with intestinal motor dysfunctions in subjects with CD and that these are reversible, something similar may occur in patients with NCGS. Nevertheless, further investigations are required in this regard.

Although our study is innovative and the findings are thought-provoking, we must acknowledge some limitations. Firstly, it is a study with a small sample size, as conducting intervention studies with a GFD before and after is complex. Nevertheless, it is noteworthy that our patients exhibited good adherence to the treatment throughout the 4 weeks of the study. Another limitation is the absence of a healthy control group, as seen in other similar studies. However, we consider the most relevant statistical comparisons to be the intervention before and after, where each subject served as their own control. It is also possible that some NCGS patients had previously attempted or been on a GFD. However, one of the inclusion criteria was a gluten challenge to confirm the diagnosis, according to what is recommended by the Salerno criteria. Lastly, the study assesses the effect of the GFD for only 4 weeks, and longer-term studies are needed, considering that histological recovery in patients with celiac disease can take 6 to 12 months.

## 5. Conclusions

In conclusion, this study demonstrates, for the first time using WMC, that a GFD improves intestinal and colonic transit, as well as contractility, in patients with CD. Similarly, albeit to a lesser extent, a gluten-free diet has a beneficial effect on the intestinal motility of patients with NCGS.

## Figures and Tables

**Table 1:** Clinical characteristics of the patients.

Clinical Characteristics	Celiac Disease	Non-Celiac Gluten Sensitivity
Gender (*n*=)		
Male	1	2
Female	11	10
Age (mean, standard deviation)	39 ± 12 years	31 ± 5 years
Body mass index (mean, standard deviation)	21.2 ± 1.9 kg/m^2^	23.8 ± 2.1 kg/m^2^
Symptoms (*n*, %)		
Bloating	12 (100%)	12 (100%)
Abdominal pain	10 (83%)	10 (83%)
Frequent bowel movements	8 (67%)	4 (33%)
Nausea	6 (50%)	3 (25%)
Borborygmi	5 (42%)	2 (17%)
Diarrhea	4 (33%)	2 (17%)
Constipation	2 (17%)	3 (25%)
Headache	2 (17%)	7 (58%)
Fogginess	4 (33%)	4 (33%)
Muscle pain	2 (17%)	6 (50%)
Marsh–Oberhüber classification (*n*, %)		
0	0	12 (100%)
1	0	0
2	5 (42%)	0
3Aa	4 (33%)	0
3B	3 (25%)	0
3C	1 (8%)	0

**Table 2:** Comparison of gastrointestinal motility parameters before and after 4 weeks of a gluten-free diet (GFD) in celiac disease and non-celiac gluten sensitivity patients.

Motility Parameters	Celiac Disease (*n* = 12)		Non-Celiac Gluten Sensitivity (*n* = 12)	
	Before	After 4 Weeks of GFD	Before	After 4 Weeks of GFD
Transit time (minutes)				
Gastric	156 ± 38	216 ± 48	183.6 ± 60	186 ± 54
Small bowel	252 ± 39	196 ± 27 *	264 ± 41	181 ± 18 *
Colonic transit	2150 ± 1020 ^&^	1450 ± 348 *	1278 ± 452	1139 ± 365
Whole gut	2394 ± 960 ^&^	2104 ± 660	1672 ± 429	1577 ± 412
Small bowel motility parameters				
Pressure maximum (mmHg)	109 ± 23	198 ± 17 *	116 ± 28	162 ± 28 *
Mean peak amplitude (mmHg)	2.4 ± 0.8	3.3 ± 0.4 *	2.8 ± 0.6	3.0 ± 0.7
Contractions per minute (number)	1.68 ± 1.4	3.74 ± 1.3 *	3.63 ± 0.9	5.2 ± 2.3 *
Motility index ^a^	136 ± 32	206 ± 24 *	160.5 ± 50	222 ± 41 *
Colon motility parameters				
Pressure maximum (mmHg)	98 ± 19	173 ± 41 *	107 ± 32	132 ± 47
Mean peak amplitude (mmHg)	3.1 ± 1.8	4.1 ± 1.9	4.2 ± 1.9	4.7 ±1.3
Contractions per minute (number)	1.9 ± 0.7	3.1 ± 0.8 *	2.1 ± 0.8	2.7 ± 0.9
Motility index ^a^	154 ± 61	208 ± 32 *	197 ± 52	224 ± 68
pH median				
Gastric	2.1 ± 0.5	1.9 ± 0.8	1.9 ± 0.3	1.8 ± 0.5
Small bowel	7.3 ± 0.6	6.9 ± 1.1	7.2 ± 0.8	7.1 ± 0.8
Colonic	6.8 ± 0.8	6.7 ± 0.9	6.9 ± 1.1	6.9 ± 0.7

^a^ Motility index is a composite parameter that incorporates both contraction frequency and amplitude and is calculated as ln (sum of amplitudes × number of contractions + 1). ^&^ *p* < 0.05 comparison between CD and NCGS. * *p* < 0.05 comparison before and after gluten-free diet. Continuous variables with normal distribution, Welch Two-Sample *t*-test; paired *t*-test. Continuous variable with non-normal distribution, Wilcoxon rank sum test.

## Data Availability

The data presented in this study are available on request from the corresponding author.
